# Traditional Chinese medicine versus regular therapy in Henoch-Schönlein purpura nephritis in children: study protocol for a randomized controlled trial

**DOI:** 10.1186/s13063-019-3484-3

**Published:** 2019-08-29

**Authors:** Ying Ding, Xia Zhang, Xianqing Ren, Wensheng Zhai, Liyun He, Jianping Liu, Chen Yao, Shanshan Han, Long Wang

**Affiliations:** 1 Pediatric Kidney Disease Center, the First Affiliated Hospital of Henan University of TCM, Zhengzhou, China; 2 Clinical Evaluation Department, China Academy of Chinese Medical Sciences, Beijing, China; 3 Evidence-Based Medicine Center, Beijing University of TCM, Beijing, China; 4 Medical Statistics Department, Peking University First Hospital, Beijing, China

**Keywords:** Henoch-Schönlein purpura nephritis, Traditional Chinese medicine, Tripterygium glycosides, Study protocol

## Abstract

**Background:**

Henoch-Schönlein purpura nephritis (HSPN) is the most common secondary glomerular disease in children. Currently, the treatment for HSPN is always selected based on the Kidney Disease Improving Global Outcomes guidelines; however, this approach may lead to undertreatment, especially in patients with persistent proteinuria that does not reach nephrotic levels and/or hematuria and those with a pathological classification between grades 1 and 3 according to the International Study of Kidney Disease in Children. This study was performed to evaluate the curative effect and safety of a traditional Chinese medicine (TCM) integrated treatment program in this type of HSPN.

**Methods:**

This multicenter, open-label, large-sample, randomized controlled trial was performed in China and included 500 children with HSPN exhibiting mild pathological patterns. The treatment group to control group ratio was 2:1, and each group was further stratified into two types, light and heavy, according to urinary protein quantification and pathological type. The treatment group received tripterygium glycosides (TGs), tanshinone IIa sodium sulfonate injection, and Chinese herbs selected based on syndrome differentiation in TCM. The heavy and light subgroups received treatment courses and dosages of TG. In the control groups, the light group received benazepril hydrochloride tablets, low molecular weight heparin calcium injection, dipyridamole tablets, and a Chinese medicine placebo, while the heavy group received the same treatment plus prednisone. All groups were treated for 3 months and then followed up for 9 months. The efficacy and safety of the treatments were then evaluated among the groups.

**Discussion:**

Currently, few treatments are available for HSPN patients with mild pathological patterns indicating light to moderate proteinuria and/or hematuresis. In this large-sample study, we provide a new approach for HSPN that includes an integrated treatment program that incorporates TCM.

**Trial registration:**

Clinical Trials.gov, NCT03591471. Re-registered on 19 July 2018.

**Electronic supplementary material:**

The online version of this article (10.1186/s13063-019-3484-3) contains supplementary material, which is available to authorized users.

## Background

Henoch-Schönlein purpura nephritis (HSPN) is one of the most common secondary glomerular diseases in children (78.9%) [1]. Approximately 30–50% of patients diagnosed with HSP develop HSPN, with risk depending on clinical manifestations [2, 3]. Although most patients with HSPN have a good chance of achieving a recovery, approximately 10–20% of patients with moderate to heavy proteinuria are at risk of kidney failure [4–6]. Therefore, it remains necessary and important to obtain an early diagnosis, to implement the right treatment strategy, and to perform long-term follow-up [7].

HSPN presents with diverse clinical manifestations, including microscopic hematuria, microalbuminuria to massive proteinuria, nephrotic syndrome (NS), acute rapidly progressive glomerulonephritis (RPGN), and acute renal insufficiency (ARI). There are also six pathological grades that are differentiated according to the International Study of Kidney Disease in Children (ISKDC) classification [8]. The current treatment strategy for HSPN is mainly based on the Kidney Disease Improving Global Outcomes (KDIGO) guidelines [9]: angiotensin-converting enzyme inhibitors (ACEIs) and/or angiotensin receptor blockers (ARBs) are recommended for use in patients with HSPN with persistent proteinuria ranging from 0.5 to 1 g/day/1.73 m^2^; while for those with persistent proteinuria (> 1 g/day/1.73 m^2^) after treatment with ACEI/ARBs and a glomerular filtration rate above 50 ml/min/1.73 m^2^, a 6-month course of corticosteroids should be used. In patients with clinical features such as massive proteinuria, NS, or RPGN or with an ISKDC grade above IIIb, we administer both steroids (prednisone and methylprednisolone) and immunosuppressive agents (cyclophosphamide, azathioprine mycophenolate mofetil, and calcineurin inhibitors). However, the KDIGO guidelines for treatment of HSPN are graded with low strength and very low quality of evidence (the recommendations were graded 2D), which were for the large part extrapolated from adult trials in IgA nephropathy, not HSPN. In addition, treatment with platelets and anticoagulants remains controversial, but the evidence-based guideline in China indicates that the addition of anticoagulants and/or antiplatelet aggregation agents could also be used to treat HSPN [10].

It is widely believed that the prognosis of HSPN depends on the severity of kidney injury, with more crescents indicating a worse outcome. Otherwise, there is a lack of high-quality data to inform treatment decisions in any subtype of HSPN, particularly in those with low-grade proteinuria or less severe pathologic classification. However, a recent study explored long-term renal prognoses in patients with grade II and no crescents, and found that 25% of these individuals retained proteinuria and thus were at continued risk of developing chronic renal failure over time [11]. Some experts have noted that the current treatment strategy for HSPN is insufficient if only the KDIGO guidelines are followed, especially in patients with mild types of lesions [11]. There are two probable reasons for this view. First, it is possible that acute glomerular inflammation previously occurred but was not treated in an accurate or timely manner. Second, even patients with minimal renal symptoms can progress to a manifestation similar to IgAN, in which proteinuria is slowly progressive and NS is less frequent [12].

It is therefore necessary to seek alternative and supplementary therapies to treat HSPN exhibiting a mild pathological pattern. Finally, we found that traditional Chinese medicine (TCM) therapy represents a new method for treating patients in this situation.

Tripterygium glycoside (TG) is the active ingredient in triptolide and a patented TCM derived from *Tripterygium wilfordii* Hook. It is sometimes used as a type of immunosuppressant in children with HSPN. A meta-analysis including 46 RCTs reported that the total effective rate (reduction of clinical symptoms and proteinuria/urinary occult blood) was better in the TG group than in a conventionally treated glucocorticoid (GC) group (RR = 1.36, 95% CI = 1.14 to 1.62; RR = 1.30, 95% CI = 1.16 to 1.46), and the time required for proteinuria and hematuria disappearance was shorter in the TG group than in the conventional treatment GC group by an average of 9 days (95% CI = – 11.99 to − 6.01; MD = – 12.00, 95% CI = – 16.13 to – 7.87) [13].

Tanshinone IIA is the main component of danshen (*Salvia miltiorrhiza*). Two studies [14, 15] reported that the therapeutic effects were better in a group treated with tanshinone IIA sulfonic acid sodium injection (TIIa) combined with GC than in the group treated with GC alone. Two other studies [16, 17] also found that TIIa combined with TG produced a higher level of decline in D-dimer and fibrinogen (FIB), and a longer prothrombin time (PT) at 4 weeks and 12 weeks than were observed in a TG group.

Qingrezhixue granule is a type of hospital preparation based on related TCM therapy that was first proposed by Professor Ding Ying and is made by the Affiliated Hospital of Henan University of TCM (Patent No. 2018 1011 8156.8). Each packet of granules contains the following drugs: Dihuang (*Rehmanniae radix*), Mudanpi (Cortex Moutan), danshen (*S. miltiorrhiza*), hanliancao (*Eclipta alba*), Chishao (*Radix paeoniae* Rubra), Sanqi (*Panax notoginseng*), xiaoji (*Herba cirsii*), and qiancao (Radix Rubiae). Unlike modern medicine, TCM is based on syndrome differentiation and is determined according to clinical manifestations and physical conditions. For example, the Professor Ding-promoted “primary syndrome” and “secondary syndrome” system classifies five TCM patterns of syndromes: wind heat complicated with blood stasis, blood heat complicated with blood stasis, dampness heat complicated with blood stasis, yin deficiency complicated with blood stasis, and both qi and yin complicated with blood stasis [18]. Qingrezhixue granules play a key role in clearing heat and cooling blood, thereby promoting blood circulation and removing blood stasis. Other herbs can also be added if necessary according to the type of syndrome.

We achieved good results by combining these comprehensive treatment programs with TCM. In our previous multicenter randomized controlled study performed in children with HSPN with proteinuria and hematuria, and renal pathology between grade I and grade III (crescents < 25%) by ISKDC classification (172 cases), we designed a 12-week course of treatment with no follow-up and found that, compared to patients treated with routine therapy (GC + benazepril LMWHC + dipyridamole), the TCM-treated group (TG + Xiangdan injection + Qingrezhixue granules) exhibited better curative effects in alleviating proteinuria (χ^2^ = 9. 5585, *P* = 0. 0227; χ^2^ = 15. 4872, *P* = 0. 0014) at 4 and 8 weeks, and reduced RBC in urine (χ^2^ = 7. 9638, *P* = 0. 0468) at 8 weeks, without any serious adverse reactions [19]. Based on a preliminary study, we further optimized the design scheme, enlarged the sample size, amplified the applicable clinical syndrome types, and divided the patients into two groups with two subgroups each according to the level of proteinuria. The different treatments were compared to evaluate the efficacy and safety of a TCM stepped care program in HSPN.

## Methods/design

### Study aim

Because regular therapies are currently insufficient, the aim of this study was to provide an alternative TCM program for children with HSPN with hematuria associated with proteinuria and isolated proteinuria. The study aimed to provide an appropriate reference dose of TG tablets for children through this comprehensive research program of TCM.

### Study design

**Fig. 1:**
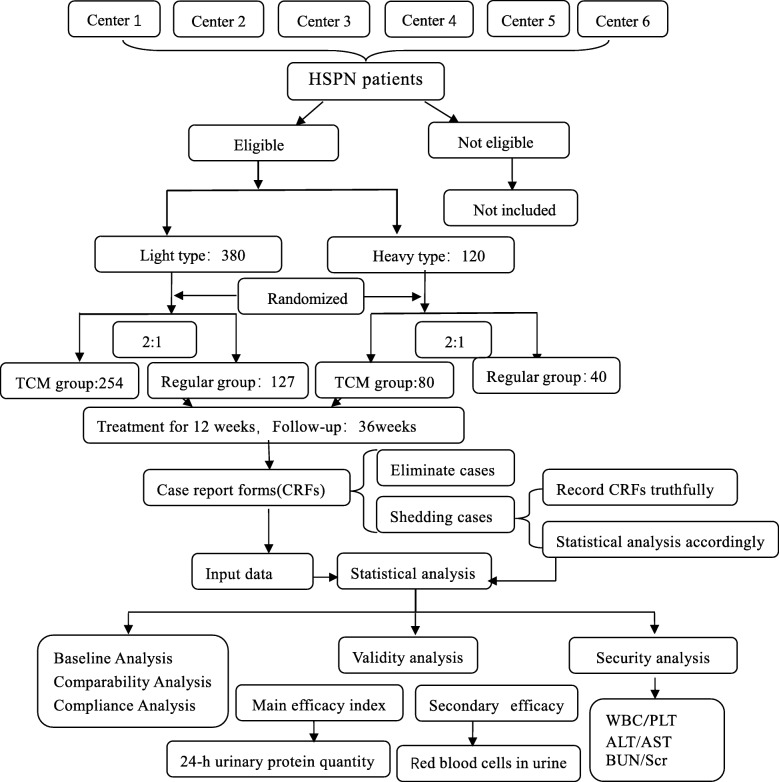
Flow diagram

This study will be a stratified, randomized, multicenter, open-label controlled prospective trial. The flow diagram is shown in Fig. 1.

### Study setting

The patients will be recruited in six class III grade I hospitals, including one sponsor (the First Affiliated Hospital of Henan UTCM) and five collaborator (Peking University First Hospital, Children’s Hospital of Fudan University, Children’s Hospital of Shanghai, Affiliated Hospital of Chengdu UTCM, and Affiliated Hospital of Yunnan UTCM) institutions. All included cases must be hospitalized.

As a responsible party, the 450-bed pediatrics department of the First Affiliated Hospital of Henan UTCM is a national key specialty department focused on HSPN. Additionally, the department has rich experience in treating various types of HSPN using combinations of TCM and western medicine, especially with regard for TG. Annually, approximately 20,000 outpatients visit, and approximately 2300 inpatients are hospitalized in the pediatric nephropathy and purpura wards. In addition, the open access of the pediatric renal pathology laboratory is convenient for patients.

The conditions described lay a solid foundation for the feasibility of this trial.

### Participant recruitment

We expect to recruit 500 eligible patients from seven hospitals in different regions of China. Parental consent will be obtained for all enrolled patients. All enrolled patients must be hospitalized in the first 2 weeks of treatment. No further recruiting measures will be taken, and no individuals with a direct relationship to the researchers, such as students/staff of the hospital or close relatives, will be included. Patient enrollment started in September 2014 and is scheduled to end in December 2018.

### Inclusion criteria

1. Clinical manifestation as proteinuria at non-nephrotic levels with or without hematuria in HSPN.
2. In children with HSPN, total urine protein at 24 h higher than 500 mg or 25 mg/kg or lower than 50 mg/kg or 3.5 g resulting in classification as light type or heavy type, respectively, according to the amount of proteinuria. Patients are biopsied if presenting 24-h protein above 1 g/day/1.73 m^2^. The renal biopsy classification can be used as a reference, and the results of urinary protein quantification (heavy vs light) are used as the basis for classification when these two criteria are conflicting (details presented in Table 1):

- Light HSPN: total urine protein at 24 h between 25 and 35 mg/kg, a renal pathological grade less than II, or IIIa with a ratio of crescents, loop necrosis, and adhesion of glomerular and renal capsules less than 10%.
- Heavy HSPN: total urine protein at 24 h between 35 and 50 mg/kg, a renal pathological grade of IIIb or IIIa and a ratio of crescents, loop necrosis, and adhesion of glomerular and renal capsule higher than 10%.

3. If undergoing kidney biopsy, a renal pathological grade between I and III based on the ISKDC classification with a ratio of crescents, loop necrosis, and adhesion of glomerular and renal capsules lower than 20%.
4. Treated with any of five TCM syndrome differentiation types, including wind heat complicated with blood stasis, blood heat complicated with blood stasis, yin deficiency complicated with blood stasis, both qi and yin deficiency complicated with blood stasis, and damp heat with blood stasis.
5. Age 2–18 years.
6. Disease onset within 2 months.
7. Abnormal urine tests for more than 1 week.
8. No use of GC and immunosuppressors, such as TG, cyclophosphamide, or mycophenolate mofetil, due to an abnormal urine test.
9. The patient or their statutory guardian agrees to participate and provides informed consent.

**Table 1:** The subtype classification in this study

Type	24-h protein (main reference)	Biopsy (secondary reference)
Light	25 mg/kg (or 500 mg)–35 mg/kg	A renal pathological grade less than II, or IIIa with a ratio of crescents, loop necrosis, and adhesion of glomerular and renal capsules < 10%
Heavy	35 mg/kg–50 mg/kg (or 3.5 g)	A renal pathological grade of IIIb or remains IIIa with a ratio of crescents, loop necrosis, and adhesion of glomerular and renal capsule > 10%

Note: Results of 24-h protein are used as the main basis for classification (heavy vs light) when these two criteria are conflicting

### Exclusion criteria

1. Age < 2 years or > 18 years.
2. Nephritis not caused by HSP.
3. Persistent hypertension, renal insufficiency, or azotemia.
4. If undergoing kidney biopsy, a renal pathological grade above IV (including IV) according to ISKDC guidelines with a ratio of crescents, loop necrosis, and adhesion of glomerular and renal capsules higher than 20%.
5. Allergic to the treatment medicine.
6. Other clinical classifications, such as NS, RPGN, and ARI.

### Intervention

The participants will be divided into two groups: an experimental group treated with TCM and a control group receiving regular treatment. In each arm, light type and heavy type HSPN will be reclassified based on the level of proteinuria and biopsy results. Eligible patients will be treated and observed for a total of 48 weeks. The first 12 weeks comprise the drug therapy stage, and the next 36 weeks comprise a follow-up observation stage.

1. Treatment stage (0–12 weeks).

- TCM group.

TG: for light type HSPN, the initial dosage is 1.5 mg/kg/day (maximum of 90 mg) for the first 4 weeks. For heavy type HSPN, the initial dosage is 2 mg/kg/day for the first 2 weeks and is reduced to 1.5 mg/kg/day (maximum of 90 mg) for the following 2 weeks. Both groups will continue to receive treatment for another 8 weeks at a dosage of 1 mg/kg/day taken orally 2–3 times per day for a total of 12 weeks.

Tanshinone IIa sodium sulfonate injection (TIIa): an intravenous drip of TIIa administered at a dosage of 1 mg/kg/day (maximum of 50 mg) in combination with 100–250 ml of a 5% glucose solution (GS) for 2 weeks.

TCM based on syndrome differentiation: in TCM-treated patients, Chinese herbs will be administered as Qingrezhixue granules according to syndrome differentiation for 12 weeks. The specific TCM treatments are presented in Table 2.

b) Regular group.

- GC: for severe type HSPN, prednisone will be given orally at 1.0 mg/kg/day for 12 weeks. In the first 4 weeks, GC is given at 1.0 mg/kg/day (maximum dose should not exceed 30 mg). The initial dose (1.0 mg/kg/day) should be reduced by 5 mg every other day for 4–8 weeks, and the dose should be reduced by 5–10 mg every week for 8–12 weeks. GC is not necessary and will not be administered for light type HSPN.
- LMWHC injection: both types will be given LMWHC via a subcutaneous injection at 100u/kg/d once a day for the first 2 weeks.
- Benazepril hydrochloride tablets (Lotensin): both types will be given Lotensin at 5–10 mg/day orally once per day for 12 weeks.
- Dipyridamole tablets (Persantine): both types will be given Persantine at 3 mg/kg orally three times per day for 12 weeks.
- Chinese medicine placebo: to increase compliance by patients seeking TCM treatment in Chinese hospitals, one kind of granule placebo similar to Qingrezhixue granules will be used in the control group, and there is no additional placebo used to blind for the other components of the TCM intervention (TG tablets and tanshinone IIa injections).
  Changes in proteinuria and hematuria and liver and kidney function will be monitored during treatment, as shown in Table 3.

**Table 2:** Herb addition based on traditional Chinese medicine syndrome differentiation

Yin deficiency complicated with blood stasis Add Chinese herbs as Qingrezhixue granules: *Rhizoma anemarrhenae*, *Cortex phellodendri*, *Rhizoma polygonati*	
Main symptoms of yin deficiency	Red tongue with less or no moss and a dark red throat, a low-grade fever with night sweat, sparse purpura
Secondary symptoms of yin deficiency	Feverishness in the palms and soles, dry mouth with thirst, a weak and rapid pulse
Symptoms of blood stasis	Dark purple tongue or bruises on tongue body; varicose veins or blood stasis in sublingual veins; fixed pain or intense pain causing an aversion to touch; thrombogenesis; dark or blue color (cyanosis) in the face, mouth, or gums, around the eyes, or in the lips and extremities; sluggish or intermittent pulse; hematuresis, epistaxis, ecchymosis, or tarry stool; dark and dull complexion; laboratory examination indicating blood circulation stasis
Diagnostic criteria	2 main symptoms of yin deficiency (or 1 main + 2 secondary symptoms) + 1 main symptom of blood stasis
Blood heat complicated with blood stasis Add Chinese herbs as Qingrezhixue granules: ComuBuball, Radix arnebiae seu lithospermi	
Main symptoms of blood heat	Excessive skin petechiae or bright red bruises located close to each other, anxiety, dry mouth, and thirst
Secondary symptoms of blood heat	Red tongue with yellow moss, strong and rapid pulse, constipation
Symptoms of blood stasis	Red tongue with yellow moss, strong and rapid pulse, constipation
Diagnostic criteria	2 main symptoms of blood heat (or 1 main symptom + 2 secondary symptoms) + 1 main symptom of blood stasis
Wind heat complicated with blood stasis Add Chinese herbs as Qingrezhixue granules: *Lonicera japonica*, Fructus Forsythiae, radix platycodonis, mint, bamboo leaf, *Schizonepetae*, Burdocks	
Main symptoms of wind heat	Bright red throat, fever, yellow mucus, small or itchy rash
Secondary symptoms of wind heat	Red tongue with thin yellow or white moss, light and rapid pulse, aversion to wind, cough
Symptoms of blood stasis	Red tongue with thin yellow or white moss, light and rapid pulse, aversion to wind, cough
Diagnostic criteria	2 main symptoms of wind heat (or 1 main symptom + 2 secondary symptoms) + 1 main symptom of blood stasis
Both qi and yin deficiency complicated with blood stasis Add Chinese herbs as Qingrezhixue granules: Astragalus, Radix pseudostellariae, Glossy Privet Fruit, *Eclipta alba*, Rhizoma polygonati	
Main symptoms of qi deficiency	Weak and easily tired, bad appetite or thin stool, susceptible to cold or diarrhea
Secondary symptoms of qi deficiency	Pale complexion, pale tongue with thin moss or thin and fat tongue with teeth marks, thin and weak pulse
Symptoms of yin deficiency/blood stasis	Pale complexion, pale tongue with thin moss or thin and fat tongue with teeth marks, thin and weak pulse
Diagnostic criteria	2 main symptoms of qi deficiency (or 1 main symptom + 2 secondary symptoms) + 2 main symptoms of yin deficiency (or 1 main symptom + 2 secondary symptoms) + 1 main symptom of blood stasis
Dampness heat complicated with blood stasis Add Chinese herbs as Qingrezhixue granules: *Scutellaria baicalensis*, Cogongrass rhizome, Herba pyrrosiae	
Main symptoms of dampness heat	Yellow complexion, dark urine or sticky stool, yellow greasy coating on the tongue
Secondary symptoms of dampness heat	Skin sores (e.g., furuncles), bitter taste and bad smell in the mouth, rapid and weak pulse
Symptoms of blood stasis	Skin sores (e.g., furuncles), bitter taste and bad smell in the mouth, rapid and weak pulse
Diagnostic criteria	2 main symptoms of blood heat (or 1 main symptom + 2 secondary symptoms) + 1 main symptom of blood stasis

**Table 3:** Observation protocol to be followed during treatment and follow-up

Observed indicator/week		Treatment stage (week)								Follow-up stage (week)								
		0	1	2	4	6	8	10	12	16	20	24	28	32	36	40	44	48
Main	24-h urinary protein quantity	×	×	×	×	×	×	×	×	×	×	×	×	×	×	×	×	×
Secondary	Urine red blood cells	×	×	×	×	×	×	×	×	×	×	×	×	×	×	×	×	×
Security	Routine blood tests	×	×	×	×	×	×	×	×	×	×	×	×	×	×	×	×	×
	Hepatic and renal function	×	×	×	×		×		×		×		×		×		×	×

2. Follow-up stage (12–48 weeks).

The 12-week treatment stage will be followed by a 36-week follow-up stage. The main aim of the follow-up is to monitor a series of laboratory indicators and determine the disease recurrence rate. The details are presented in Table 2.

In patients who meet the clinical control standards after 12 weeks of treatment, medication will be stopped and the patients will begin follow-up. The clinical control standards mean that RBC counts and 24-h protein in urine are at normal levels, and urine routine examinations are negative for protein and occult blood. For those patients who do not meet the clinical control standards by 12 weeks, other treatment options could be appropriately selected in the professional judgment of the clinicians. The clinicians who are evaluating the follow-up laboratory results (and determining whether there is a relapse or whether the patient has reached clinical control standards) are not blinded to the patient’s treatment assignment. The criteria for efficacy evaluation are presented in Table 4.

**Table 4:** Criteria for efficacy evaluation

Primary outcome	Clinical control rate	Significant efficiency	Effective rate	Ineffective rate
PRO in urine	Negative	PRO decreased by 2+	PRO decreased by +	PRO decreased less than +
	24-h urinary protein quantity is normal	24-h urinary protein quantity reduced > 50%	24-h urinary protein quantity reduced between 30 and 50%	24-h urinary protein quantity reduced < 30% or no change
RBC in urine	U-OB: negative	U-OB: RBC reduction ≥ ++/HP	U-OB: RBC reduction ≥ +/HP	U-OB: no improvement or aggravation in RBC
	RBC ≤ 5/HP	RBC count decreased By > 50%	RBC count decreased between 30 and 50%	RBC count decreased < 30% or no change
Total therapeutic effect	Meet all these requirements	Meet all these requirements	Meet all these requirements	Meet all these requirements

*RBC* red blood cells, *U-OB* urine occult blood, *PRO* Urinary protein, *HP* high power microscope

If the disease relapses during follow-up, the details of the recurrence will be recorded and the appropriate treatment administered in a timely manner while follow-up is completed. Other treatment options are available to patients who do not meet the clinical control standards, and it will be necessary to record any decisions and proceed with the follow-up stage.

### Abscission or termination of allocated interventions

Any subject who does not complete the observation period as specified in the protocol should be recorded as a case of abscission unless the patient’s symptoms disappeared before treatment.

The following are possible reasons for abscission or termination:

1. Poor drug compliance will be considered if the medication compliance test result is less than 80%. Compliance = (total number of pills taken / prescribed pills) × 100%.
2. Consumption of drugs that are not permitted during the clinical study, including other hormones and immunosuppressants not mentioned in the trial such as cyclophosphamide, mycophenolate, and so on.
3. Incomplete information due to failure to complete the entire course of treatment for any reason.
4. Serious adverse events or complications making it inappropriate to continue the trial and resulting in the discontinuation of treatment.

The following measures should be taken:

1. Attempt to contact the subject to ask for an explanation for abscission or termination and record the last time the patient took the medicine and completed an assessment.
2. In the case of withdrawal from the test due to anaphylaxis, adverse reactions, or ineffective treatment, appropriate treatment measures should be actively taken.
3. An abscission case form should be completed that explains the cause in detail.
4. Statistical analyses, including analyses of intention-to-treat (ITT) and safety set (SS), will be performed on abscission cases as appropriate. If the abscission rate exceeds 5%, the reason for abscission will be explained in detail.

Termination of study cases may occur for the following reasons:

1. In children with light type HSPN, if the 24-h urinary protein quantity reaches the heavy type (35–50 mg/kg) during the second week of treatment, the patient may be considered an invalid case and potentially be randomly regrouped as heavy type HSPN; it should be noted that for cases invalid in light type, the outcome will be included in the intention-to-treat analysis instead of excluding these patients. For participants who have already been re-randomized as heavy type, the data will be analyzed separately. Sensitivity analyses with and without these particular patients who may have crossed over between interventions will also be performed.
2. In patients with serious adverse events or no therapeutic effects after 4 weeks of treatment, the trial should be discontinued at the doctor’s discretion; they are no longer followed and the outcome is not included in the analysis.
3. Patients who are unwilling or unable to continue the clinical trial for any reason.

### Adverse events and serious adverse events

Every adverse events (AE) that occur during the trial will be faithfully recorded and evaluated for a possible association between AEs and the experimental drugs in accordance with the Guidelines for Clinical Research of New Chinese Medicine Drugs (Trial), a standard designated by the pharmaceutical adverse reaction monitoring center of the Ministry of Health. Necessary measures will be immediately taken to protect the safety of the patients and to determine whether to stop observation.

If any adverse events occur during the study, the investigator should take necessary measures according to the patient’s condition with time. In the case of mild adverse events not affecting the subject’s health, no special treatment or symptomatic treatment is required; for moderate adverse events, the trial should be discontinued and targeted treatment should be given to the subjects.

When serious adverse events (SAEs) occur during a trial, it is extremely important to report them to the responsible units involved in the project, including the Ethics Commission, the Data and Safety Monitoring Board, and the Food and Drug Administration (FDA), within 24 h. Severe adverse events will endanger the lives of subjects, and the clinical trials should be stopped immediately and emergency treatment should be given. The units in charge of the project and the participating units should make common emergency plans for dangerous conditions such as allergic reaction, cardiac arrest, respiratory failure, heart failure, and so on.

### Outcomes

The study will be conducted over a period of 1 year (48 weeks) and will include a treatment period of 3 months (12 weeks) and an observation period of 9 months (36 weeks). In all, one primary outcome indicator, one secondary outcome indicator, and four security indexes will be analyzed:

1. Primary outcome indicator: 24-h urinary protein quantity.

Any change in total urine protein level at 24 h will be assumed to directly reflect a therapeutic effect; hence, this clinical laboratory index should be recorded every 2 weeks during the treatment period and every 4 weeks during the follow-up period (17 times in total). In addition, creatinine excretion measured vs expected for gender will be detected at the same time to verify the adequacy of 24-h urine collection indirectly.

Total urine protein at 24 h is the most important outcome. In previous studies, we found that the improvement of urinary protein of TG at week 4 and week 8 was better than that of the control group, while the difference at week 12 was not significant. Based on these subtypes, this study will further explore the difference in treatment between the two groups. Therefore, we will set 17 nodes, including the treatment period and the follow-up period, to compare the differences in urine protein between the two groups at each node, rather than only retaining the last result at 48 weeks.

2) Secondary outcome indicator: red blood cells in urine.

Compared to the primary indicator, the urine red blood cell count is considered secondary because urine red blood cells recede more slowly than protein recedes. This clinical laboratory index should be recorded at the same points during treatment and follow-up as the primary outcome indicator (17 times in total).

3) Security indexes.

- Blood system: the white blood cell count (WBC) and platelet count (PLT).
  In patients taking immunosuppressors or prednisone, there is a potential risk of abnormalities in the blood system in terms of leukocyte and platelet counts. Blood tests were performed to determine the leukocyte count 17 times during and after treatment, as already described.
- Liver and kidney function test: glutamate alanine transferase (ALT), glutamate aspartate transferase (AST), serum creatinine (Scr), and blood urea nitrogen (BUN) levels should be monitored.
  In patients using any drug, there is a potential risk of dysfunction in the liver and kidney. Because they are the main indicators used to evaluate liver and kidney function damage, blood should be drawn from the vein to perform ALT/AST and Scr/BUN tests 11 times during and after treatment.

Time frame for testing: week 0 (before treatment)/week 1/week 2/week 4/week 8/week 12 of the treatment phase and week 20/week 28/week 36/week 44/week 48 of the follow-up phase.

### Randomization

Participants will be randomized at a 2:1 ratio by the Central Randomization System of the China Academy of Chinese Medical Science, which is an online, central randomized service. Allocation concealment will be ensured, as the service will not release the randomization code until the patient has been recruited into the trial, which takes place after all baseline measurements have been completed.

The study participants are stratified by disease subtype. Both heavy type and light type will be included in each subcenter. The trained clinicians divide those children with HSPN who met the requirements into light type and heavy type according to the amount of urine protein within 24 h. After signing the informed consent, clinicians will log into the central random system according to their account password, and then click “random” under the corresponding subtype to conduct random grouping.

### Allocation

All drugs will be uniformly distributed to the subcenters by the First Affiliated Hospital of Henan University of TCM. Each drug is obtained from the same manufacturer with the same specifications according to the following details:

- Tripterygium glycoside tablets (Jiangsu Meitong Pharmaceutical Co. Ltd; SFDA approval number Z33020422, 10 mg).
- Sulfotanshinone sodium injection (Shanghai First Biochemical Pharmaceutical Co. Ltd; SFDA approval number H31022558, 2 ml, 10 mg).
- Qingrezhixue granules (hospital preparations created by Professor Ding Ying and produced at the First Affiliated Hospital of Henan University of TCM; Patent No. 201810118156.8).
- Prednisone acetate tablets (Zhejiang Xianju Pharmaceutical Co. Ltd; SFDA approval number H33021207, 5 mg).
- Benazepril hydrochloride tablets (Beijing Nuohua Pharmaceutical Co. Ltd; SFDA approval number H20000292, 10 mg).
- Low molecular weight heparin calcium injection (Tianjin Hongri Pharmaceutical Co. Ltd; SFDA approval number H20020470, 6000 U).
- Dipyridamole tablets (Linfen Qilin Pharmaceutical Co. Ltd; SFDA approval number H14020538, 25 mg).
- Chinese medicine granule and granule simulation drugs (placebo) (produced by Jiangyin Tianjiang Pharmaceutical Co. Ltd).

All manufacturers meet GMP (Good Manufacturing Practice) standards. All drugs should be kept in a dry and cool place by a specially trained person.

### Blinding

Because TCM syndrome differentiation is needed during the research process, this is an open-label trial. To improve patient compliance in patients who come to the Chinese Medicine Hospital for TCM treatment, a regular group will be treated with Chinese medicine granule simulation drugs (placebo).

### Sample size calculation

The previous clinical results showed that the clinical control rate of proteinuria was 48.6% in the TCM group and 35.7% in the western medicine group during the 4th week of treatment. Based on these results, in this study, the total sample size was calculated as 405 cases if the TCM group and the western group were included at a ratio of 2:1. The δ value between them is expected to be 15% with a significance level of α = 0.05 and an assurance of 1 – β = 90%. We will increase the sample size by approximately 20% to reach 500 cases in consideration of the possibility that some patients could miss visits due to the long illness course and follow-up time. Clinical experience indicate that heavy patients will account for approximately 25% of eligible individuals; hence, 120 patients with heavy HSPN and 380 patients with light HSPN will be included.

### Data collection and management

Case report forms (CRFs), medical reports and laboratory test reports will make up the main data source file for the subjects in this clinical trial. Data obtained from each subcenter should be regularly transferred in a timely manner to the subject undertaking unit. For all eligible CRFs, two data entry staff will independently input the contents into the database as double copies according to the requirements. During this process, if any data are in doubt, they will be entered into a “Data query table” and fed back to the clinical researchers by the data administrators. The database is locked and will be submitted to the quality control group of the “Twelfth Five-year” Plan for the National Science and Technology Program through the data management network after a data consistency check, all database modifications, and a data quality audit are completed.

### Statistical analysis

All statistical analyses in the study will be carried out by a third party, the Chinese Academy of Traditional Chinese Medicine, using SAS version 6.12, which is validated software for clinical studies. Logistic regression is used to show a difference in the prevalence of clinical control/remission in both groups, including age, gender, place of residence, parents’ educational background, and so on. For the outcome “time from positive to negative for urinary protein and urinary occult blood”, a Cox proportional hazards model is used. The incidence is described for adverse events and Fisher’s test will be used for comparison. Besides, groups and time of therapy on curative effect will be considered. If there are interaction effects between groups, simple effect analysis is performed; if not, grouping effect/time of therapy on curative effect should be considered separately.

### Quality control and quality assurance

It is necessary to take appropriate measures to minimize bias because confounding bias is a key factor that can affect the quality of the test.

We will try to select research centers in different regions and strictly follow the inclusion and exclusion criteria to reduce selection bias. Conduct unified training and assessments of participating researchers and strictly normative data will be performed to reduce measurement bias. To ensure the traceability of laboratory test results and minimize measurement bias, the normal reference values of the laboratory index of each subcenter will be attached.

Concomitant medications should be reduced as much as possible during study implementation.

In addition, the advantages and characteristics of the study will be explained to the subjects in detail before enrollment so that the patients and/or their guardians will fully understand the possible benefits, to improve compliance and to reduce shedding.

It is necessary to establish a three-grade quality control mechanism as the quality supervision organization: researchers conduct self-examination about the cases they have included, inspectors from each subcenter supervise every 4 months, and inspectors from the research group supervise each year. There will be no additional sponsorship.

A comprehensive Data and Safety Monitoring Board will be established to ensure the quality of each step during the testing process as much as possible. As an independent party, the Data Monitoring Committee (DMC) is responsible for the quality control and safety supervision of this study to ensure the safety of the subjects and the validity of the data. By reviewing the implementation, data quality, and evidence of adverse events, the trials can be terminated if the examination indicates that the cases are useless or invalid. When adverse events occur, the main investigator of each center can decide whether to suspend the observation of this case according to the patient’s condition. In addition, the DMC has the right to terminate the trial when data are found to be invalid or useless. The Spirit checklist was shown in Additional file 1.

## Discussion

According to a national cross-sectional survey on the spectrum of biopsy-proven glomerular diseases among children that was performed in China from 2004 to 2014, HSPN (13%) and lupus nephritis (9%) are the most common secondary glomerular diseases in children, and purpura nephritis (23%) is the most common pathological pattern observed in younger children (0–12 years old) [20]. It has been reported that 6–24 per 100,000 children younger than 17 years of age will develop HSP, and approximately 30–50% of patients with HSP will develop HSPN [21]. Most of these patients manifest mainly with microscopic hematuria or proteinuria, while a few exhibit macroscopic hematuria.

Because the treatment strategy for purpura nephritis that is recommended in the current guidelines is insufficient, especially in pathological types without crescents, we propose a comprehensive treatment program in which the TCM TG is used as the main drug in combination with tanshinone, an anticoagulant drug. Furthermore, in this program, TCM is based on syndrome differentiation to evaluate treatment efficacy and provide an evidence base for the treatment of purpura nephritis. The results of this study, which will include a large sample population, will provide a new option for treating HSPN via an integrated treatment program that includes TCM.

### Trial status

Patient recruitment began in 2014 and the trial was re-registered on July 19, 2018 at ClinicalTrials.gov. The programmed completion date for the recruitment was December 31, 2018. The trial is now in the statistical stage of data.

## Additional file

Additional file 1: Spirit checklist (PNG 650 kb)

## Data Availability

The data are currently unavailable.

## Abbreviations

ACEI: Angiotensin-converting enzyme inhibitor
ARB: Angiotensin receptor blocker
CRF: Case report form
GC: Glucocorticoid
HSPN: Henoch-Schönlein purpura nephritis
ISKDC: International Study of Kidney Disease in Children
KDIGO: Kidney Disease Improving Global Outcomes
LMWHC: Low molecular weight heparin calcium
NS: Nephrotic syndrome
RPGN: Rapidly progressive glomerulonephritis
TCM: Traditional Chinese medicine
TG: Tripterygium glycoside
