# Supplementary figures and images for: Traditional Chinese medicine versus regular therapy in Henoch-Schönlein purpura nephritis in children: study protocol for a randomized controlled trial
Source: Trials. 2019 Aug 29;20:538. doi: 10.1186/s13063-019-3484-3 (PMC6716925; doi:10.1186/s13063-019-3484-3)

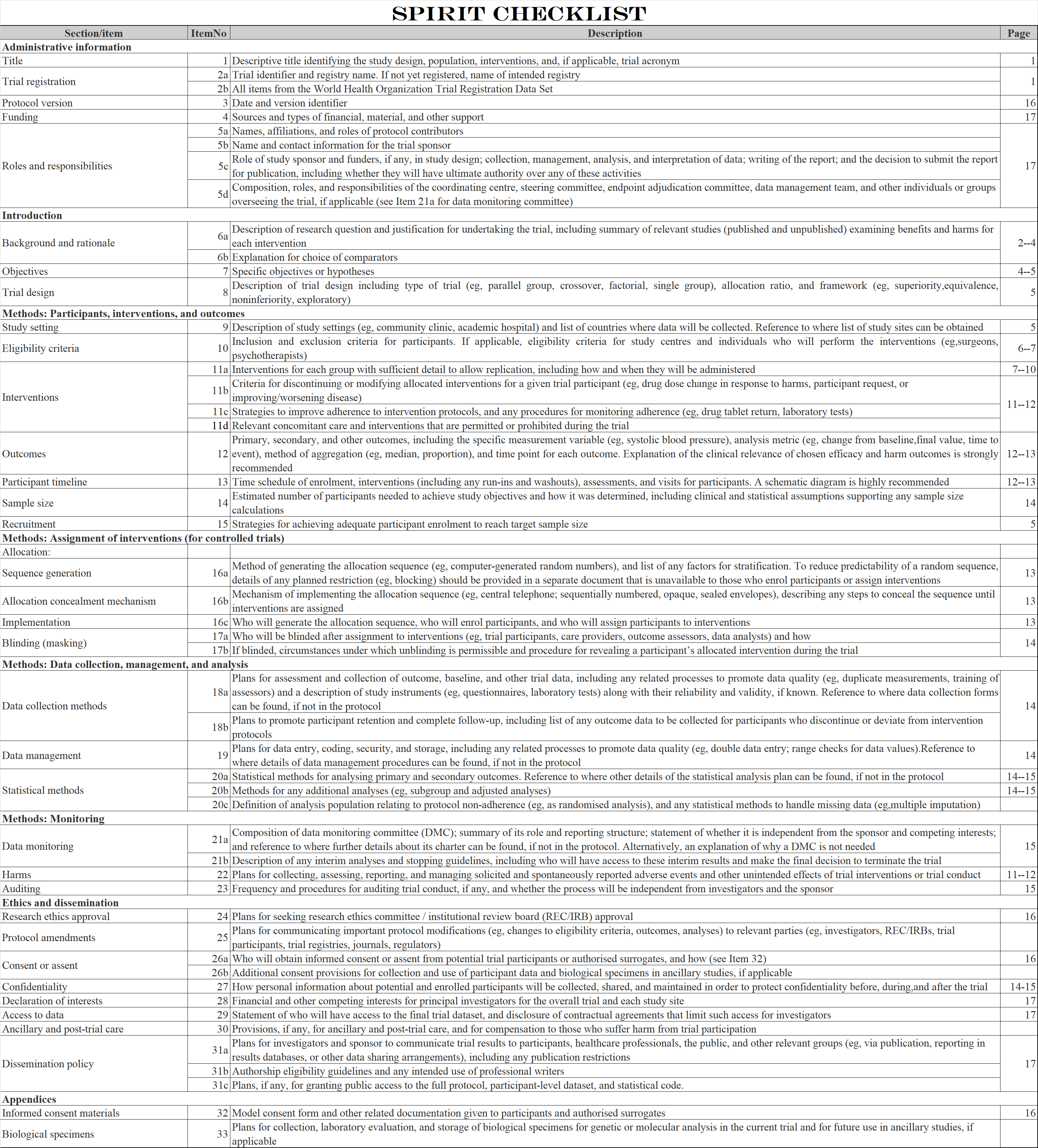

Supplement: Supplementary file 1 — Spirit checklist (PNG 650 kb) [file 13063_2019_3484_MOESM1_ESM.png]
